# Brassinosteroid Supplementation Alleviates Chromium Toxicity in Soybean (*Glycine max* L.) via Reducing Its Translocation

**DOI:** 10.3390/plants11172292

**Published:** 2022-09-01

**Authors:** Farwa Basit, Javaid Akhter Bhat, Jin Hu, Prashant Kaushik, Ajaz Ahmad, Yajing Guan, Parvaiz Ahmad

**Affiliations:** 1 The Advanced Seed Institute, College of Agriculture and Biotechnology, Zhejiang University, Hangzhou 310058, China; 2 International Genome Center, Jiangsu University, Zhenjiang 212013, China; 3 Independent Researcher, 46022 Valencia, Spain; 4 Department of Clinical Pharmacy, College of Pharmacy, King Saud University, Riyadh 11451, Saudi Arabia; 5 Department of Botany, GDC Pulwama, Srinagar 192301, Jammu and Kashmir, India

**Keywords:** foliar spray, stress tolerance, oxidative damages, soybean, metal uptake

## Abstract

Chromium (Cr) phytotoxicity severely inhibits plant growth and development which makes it a prerequisite to developing techniques that prevent Cr accumulation in food chains. However, little is explored related to the protective role of brassinosteroids (BRs) against Cr-induced stress in soybean plants. Herein, the morpho-physiological, biochemical, and molecular responses of soybean cultivars with/without foliar application of BRs under Cr toxicity were intensely investigated. Our outcomes deliberated that BRs application noticeably reduced Cr-induced phytotoxicity by lowering Cr uptake (37.7/43.63%), accumulation (63.92/81.73%), and translocation (26.23/38.14%) in XD-18/HD-19, plant tissues, respectively; besides, improved seed germination ratio, photosynthetic attributes, plant growth, and biomass, as well as prevented nutrient uptake inhibition under Cr stress, especially in HD-19 cultivar. Furthermore, BRs stimulated antioxidative defense systems, both enzymatic and non-enzymatic, the compartmentalization of ion chelation, diminished extra production of reactive oxygen species (ROS), and electrolyte leakage in response to Cr-induced toxicity, specifically in HD-19. In addition, BRs improved Cr stress tolerance in soybean seedlings by regulating the expression of stress-related genes involved in Cr accumulation, and translocation. Inclusively, by considering the above-mentioned biomarkers, foliar spray of BRs might be considered an effective inhibitor of Cr-induced damages in soybean cultivars, even in Cr polluted soil.

## 1. Introduction

Soybean (*G. max.* L.) is known as the most planted oil crop world widely, which has the capability to fix nitrogen of rhizobia and has a great impact on soil fertility [1]. Globally, soybean is considered an important source of protein for human consumption as well as animal feed [2]. The quality, yield, seed germination, growth, and biomass of soybean are adversely affected, when it is grown in heavy metals (HMs) contaminated soil [3]. Hexavalent chromium (Cr-VI) is deliberated as 1st class carcinogenic toxic element by the International Agency for Research on Cancer (IARC) [3,4]. Total chromium, including both trivalent (III), and hexavalent (VI) forms is released nearly 1.3 × 10^5^-ton worlds widely inside the soil, water, and air via numerous natural as well as anthropogenic activities which are possessing high risks to humans, plants, and animal health [4,5]. Cr is a non-essential element for plants and is present in both trivalent (III), and hexavalent (VI) forms. However, Cr (VI) is the more hazardous form of Cr in terms of toxicity, bioavailability, and mobility as compared to Cr (III) [6,7]. Plants generally uptake Cr mainly via roots, and then its small portion is further transferred to the shoots and leaves. The excessive Cr in plant tissues causes toxic effects on physiological, morphological, metabolic, biochemical, and molecular attributes [8]. Earlier investigations have revealed that Cr has inhibited the seed germination indices, plant biomass, growth, and chlorophyll pigments [7,8,9,10] as well as caused deformities of flower organs or fruits [11]. Particularly, Cr (VI) at ppm level causes a significant reduction in seed germination attributes of rice [7,12], mung bean [13], alfalfa [14], barnyard grass [15], and kidney bean [16] when Cr contents in soil reach 500 mg/kg. Furthermore, Cr also causes water retention, and nutrient uptake imbalance reduces plant photosynthetic pigments levels and destroys antioxidant enzymatic as well as non-enzymatic activities by increasing reactive oxygen species (ROS) overproduction, which further induces cellular oxidative damage [2,8,9]. Thus, the deleterious effects of Cr led to a lessening in crop yield, and quality as well [5].

Exogenous application of phytohormones is a well-known approach used to alleviate the injurious effects induced by Cr toxicity in various plant species. Brassinosteroids (BRs) are 6th class polyhydroxy steroid hormones, which are involved in plant developmental, and metabolic as well as stress mitigation attributes [6,17]. Three important BRs types Brassinolide, 24-epibrassinolide (EBL), and 28-homobrassinolide (28-HomoBL) have been widely investigated in different aspects. Previously, it is investigated that 24-EBL can minimize the HMs accumulation inside plants, enhance plant growth, chlorophyll contents, sugar, and proline contents, and modulate the antioxidant enzymatic, and non-enzymatic defense mechanism [18,19,20,21,22]. Recent documentation has demonstrated that exogenous application of BRs enhanced the photosynthetic pigments level, F*v*/F*m* values, and sugar contents by reducing the electrolyte leakage, MDA level, and Cd accumulation inside *Solanum nigrum* L. seedlings [23]. It also has been noticed that BRs improved the plant’s growth and biomass by maintaining the nutrient uptake balance and modulating the antioxidative enzyme activities in rice cultivars under Cr stress [6,24]. The exogenous application of BRs has also improved the plant biomass by elevating the enzymatic activities, and decreasing cellular oxidative injuries caused by ROS overgeneration in *Raphanus sativus* [25], and rice [12] seedlings plant. However, the BRs role in alleviating the Cr-induced phytotoxicity in soybean seedlings is least studied.

Different genes are involved in the uptake, translocation, and absorption of Cr, which may also have an essential role in the BRs-mediated Cr detoxification mechanism [21,26,27]. Few scientists have proposed that there is an independent mechanism for Cr (III), and Cr (VI) uptake in plants [28]. Sulfate (S) transporters (SULTRs) and phosphate (P) transporters are deliberated to be proficient in regulating the Cr (VI) absorbance through plant roots as a result of the structural similarity of Cr (VI) with P, and S [26,27]. Earlier studies have reported that some protein families may involve in HMs toxic elements transportation inside different plants, i.e., Zinc/iron regulatory transporter-related protein (ZIP), natural resistance-related macrophage protein (NRAMP), P-type heavy metal ATPase (HMA), and ATP binding cassette transporter (ABC), etc. [29,30,31]. Furthermore, these genes may also involve in Cr uptake, transportation, and accumulation in plants. Additionally, Phytochelatins (PCs) and homophytochelatins (hPCs) are synthesized by phytochelatins synthase family 1 ((h)PC-synthase 1, (h)PCS1), which is crucial for the detoxification of HMs toxic elements, and to enhance tolerance capability inside plants [32,33]. Though, the BRs-mediated Cr uptake, accumulation, transportation, and detoxification mechanism inside soybean cultivar is still undefined, and need to be explored.

Based on the above-mentioned discussion, the recent investigation was directed to explore the potential role of BRs toward Cr-induced stress alleviation in soybean seedlings by estimating the phenotypical, physiological, as well as metabolic biomarkers. To testify the impact of BRs with/without Cr stress on plants growth, biomass, chlorophyll contents, gas exchange indices, nutrient acquisition, inter-cellular Cr-accumulation, lipid peroxidation, ROS production as well as antioxidant enzymatic, and non-enzymatic defense machinery. In addition, the Cr stress-responsive genes expression, and BRs-mediated stress detoxification mechanism at the molecular level will be identified. The current study will highlight the developing tactics via using BRs to lower the Cr-induced phytotoxic risks for sustainable crop production.

## 2. Results

### 2.1. Effect of BRs on Seed Germination Parameters

In a current investigation, no significant difference was noted in seed germination indices germination energy (GE), germination percentage (GP), germination index (GI), vigor index (VI), and mean germination time (MGT) under control conditions. However, the GI was observed to be slightly improved by the foliar application of BRs in both soybean genotypes (Table 1), with the effect being higher in HD-19. However, the MGT was a little reduced with the application of BRs in both soybean plant types. Whereas the latest research revealed that Cr-mediated toxicity caused severe inhibition inside the seed GE, GP, GI, and VI, as well as, significantly proliferated the MGT in both soybean cultivars, especially XD-18 was more affected (Table 1). Stimulatingly, the foliar application of BRs significantly improved the above-mentioned seed germination indices under Cr toxicity in both soybean genotypes. As well, an obvious decrease in MGT was remarked by BRs treatment in both soybean varieties under Cr disclosure. Even so, the effect was more noticeable in HD-19 than in the XD-18 genotype (Table 1). Overall, the foliar exposure of BRs was significantly effective to mitigate the Cr-induced toxicity in seed germination indices of soybean.

### 2.2. Impact of Cr-Induced Stress on Phenotypical Trails of Soybean Seedlings

The visual phenotypical changings under Cr stress were observed in soybean cultivars (Figure S1). The plants treated with water (H_2_O) and foliar application of BRs without Cr-exposure were considered as the control group (CK). The sole treatment of foliar spray with BRs caused a slight increase in plant growth as compared to the plants treated with H_2_O (Figure S1). Under Cr-disclosure, a significant reduction in plant length was observed with the yellowish coloring of plant leaves in both soybean cultivars but XD-18 was being more affected relative to the HD-19. Interestingly, the foliar application of BRs improved the plant height significantly and lowered the toxic symptoms of Cr-induced stress under Cr toxicity as compared to the plants treated with alone-Cr exposure in both soybean cultivars. However, this increment in plant height with greenish leaves texture was more prominent in HD-19.

### 2.3. BRs Reduces the Chromium Stress by Improving Plant Growth, and Biomass Attributes

In a recent study, no significant variations were observed in plant growth and biomass under control conditions. The application of BRs slightly improved the plants’ biomass (fresh and dry weight), and growth (plant length) under control conditions in both soybean varieties relative to the plants treated with water (Table 1). Whereas, a remarkable reduction in plant height, fresh and dry weight was pragmatic under alone Cr-exposure to soybean genotypes, precisely XD-18 being more affected. Although, an obvious improvement in plant growth as well as biomass attributes were observed under BRs application Cr-induced toxicity in both soybean varieties, especially in HD-19 (Table 1). Generally, these outcomes demonstrated that BRs significantly minimized the Cr-induced injurious effects on plants’ biomass, and growth indices as well in both types of soybean genotypes, particularly in HD-19.

### 2.4. BRs Improves Plant Photosynthetic Traits under Chromium Stress

**Figure 1:**
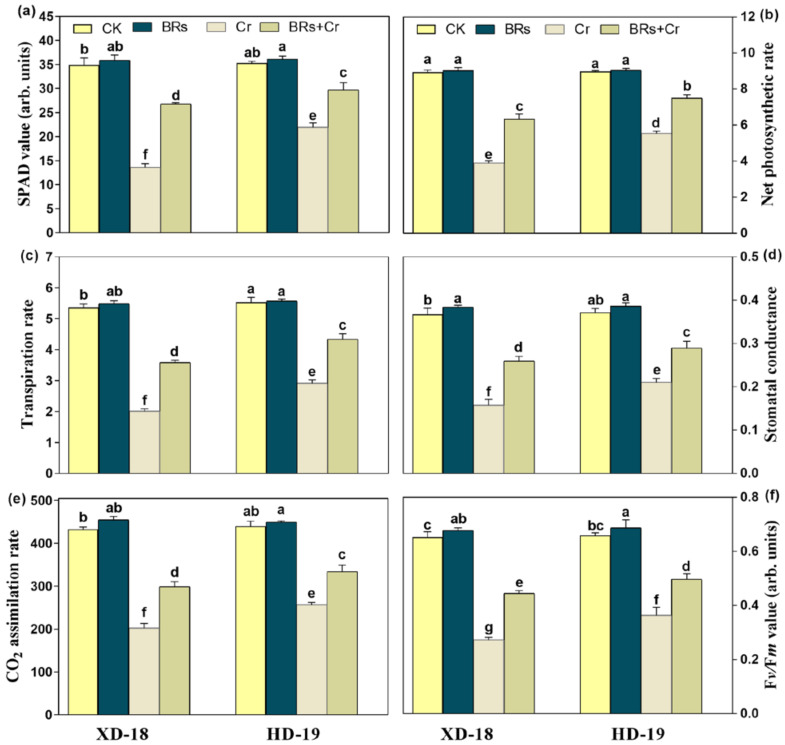
Effect of foliar applied BRs (0.01 µM) on photosynthetic attributes [(**a**) SPAD values, (**b**) Photosynthetic rate, (**c**) Transpiration rate, (**d**) Stomatal conductance, (**e**) CO_2_ assimilation rate, and (**f**) F*v*/F*m* value] in the leaves of two soybean genotypes (XD-18, HD-19) under/without chromium (Cr) (0 and 100 µM) stress. Values are the means ± SD of four independent replicates. Different alphabetical letters on bars indicate significant differences among the treatments (*p* < 0.05) by the least significant difference test.

**Figure 2:**
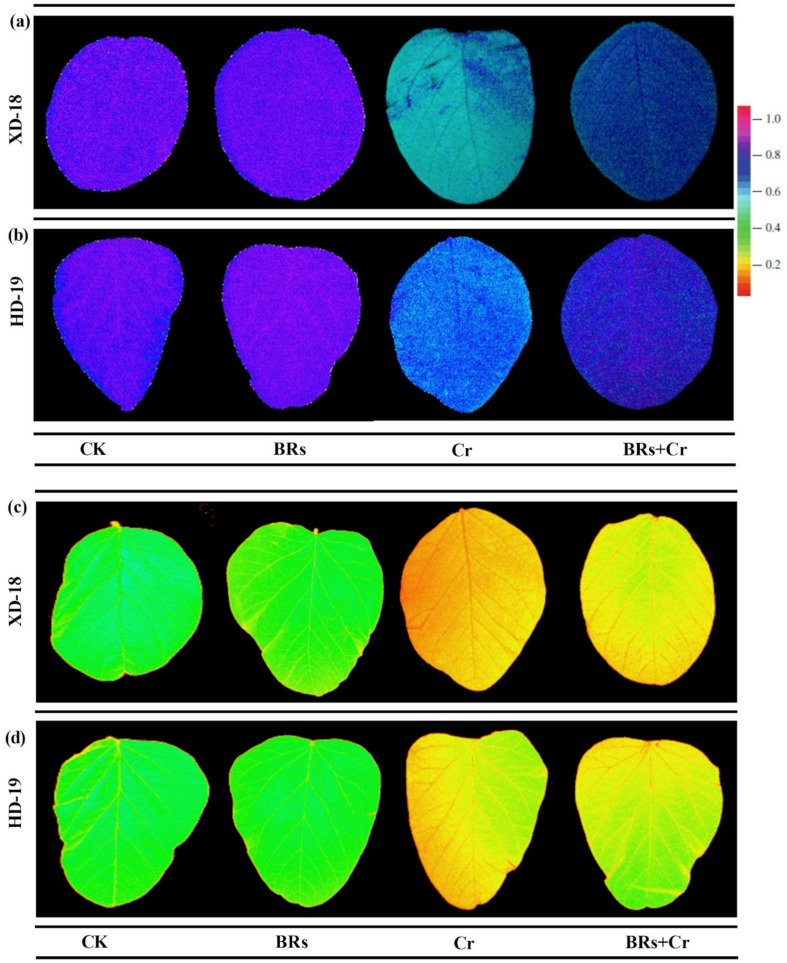
Effect of foliar applied BRs (0.01 µM) on F*v*/F*m* (**a**,**b**) and F*m* (**c**,**d**) levels in the leaves of two soybean genotypes (XD-18 and HD-19) under/without chromium (Cr) (0 and 100 µM) stress.

As expected, there was no significant difference in the values of chlorophyll contents such as SPAD, net photosynthetic rate (Pn), transpiration rate (Tr), stomatal conductance (Gs), CO_2_ assimilation rate as well as F*v*/F*m* by foliar application of BRs in comparison to the plants treated with water under control condition (Figure 1). Among Cr exposure, a significant decline was observed in the values of the above-mentioned parameters by 62.85/49.17%, 56.67/38.44%, 62.96/47.93%, 60.52/44.73%, 55.51/42.98%, and 60.29/47.05%, respectively as compared to the control in both soybean cultivars XD-18/HD-19 (Figure 1). However, BRs supplementation inhibited the Cr-induced stress damages in photosynthetic attributes under Cr toxicity by up-surging the SPAD values 27.58%/35.11%, net photosynthetic rate 25.32/39.68%, transpiration rate 32.56/42.85%, stomatal conductance 25.98/33.23%, CO_2_ assimilation rate 23.35/32.21%, and F*v*/F*m* 26.53/35.63%, correspondingly as compared to the alone treatment of Cr in both XD-18/HD-19 soybean varieties (Figure 1). Additionally, it was further verified by taking false-colored images of F*v*/F*m*, and F*m* using ImagingWin software (IMAGING-PAM, Walz, Effeltrich, Germany) (Figure 2a–d). The intensity of blue color in CK, and BRs treatments represented the higher level of F*v*/F*m* under control conditions. However, the zinc color revealed the Cr-induced toxic effect on F*v*/F*m* level under Cr stress, which was lowered by BRs treatment under Cr toxicity in both soybean cultivars (Figure 2a,b). Although, the intensity of the green color showed a greater level of Fm inside the control group (CK, and BRs without Cr stress). While, reddish color intensity demonstrated the Cr-induced stress effects on the Fm level, which was diminished by BRs + Cr treatment in both XD-18, and HD-19 cultivars shown in Figure 2c,d. These findings revealed that BRs application can significantly improve the photosynthetic level, and gas exchange parameters in different soybean varieties under a Cr-contaminated environment; particularly HD-19 was more positively affected.

### 2.5. BRs Reduces Cr Uptake and Maintains Nutrient Homeostasis under Chromium Stress

**Figure 3:**
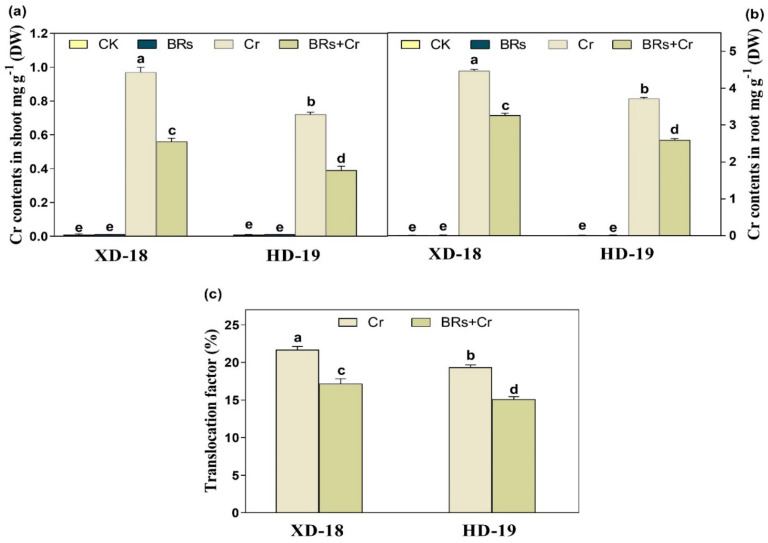
Effect of foliar applied BRs (0.01 µM) on Cr contents, and translocation factor in tissues of two soybean genotypes (XD-18 and HD-19) under chromium (Cr) (0 and 100 µM) stress. (**a**,**b**) Cr contents in shoots, and roots; (**c**) Translocation factor. Values are the means ± SD of four independent replicates. Different alphabetical letters on bars indicate significant differences among the treatments (*p* < 0.05) by the least significant difference test.

Relative to the control plants, a sharp Cr accretion was noticed in roots than shoots under the exogenous exposure of alone Cr in both soybean cultivars (XD-18, and HD-19). Both genotypes represented significantly different Cr accumulation levels, with XD-18 containing a higher Cr concentration than HD-19 (Figure 3a,b). While, the BRs supplementation significantly reduced the Cr uptake in both shoots, and roots of two various soybean cultivars. Furthermore, BRs application restricted the Cr translocation from roots to shoots, as designated by the abridged translocation factor (Figure 3c). As compared to the control treatment, Cr alone treatment significantly minimized the uptake as well as translocation of macronutrients such as Ca, Mg, Mn, Zn, and Cu. The decrease in uptake of the above-mentioned macronutrient was clearer in roots than in shoots of both soybean cultivars, specifically in XD-18 (Table 2). Whereas, the foliar application of BRs significantly maintained the nutrient balance in both soybean cultivars under Cr-induced toxicity. Inclusive, these outcomes revealed that BRs supplementation lowered the adverse effects of Cr-induced stress on both soybean genotypes by maintaining the nutrient uptake balance and restricting the Cr uptake, and translocation in both soybean varieties.

### 2.6. BRs Modulates the Electrolyte Leakage, Total Soluble Sugar, and Protein under Chromium Stress

**Figure 4:**
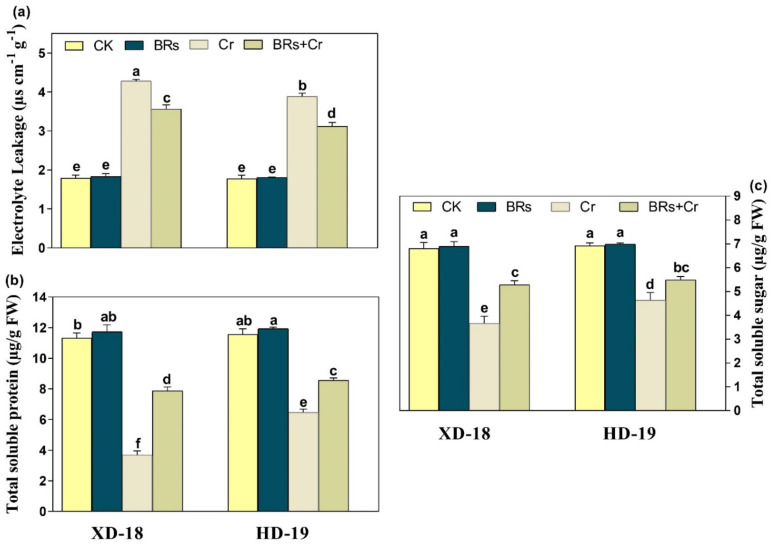
Effects of foliar-applied BRs on (**a**) electrolyte leakage (EL), (**b**) total soluble proteins, and (**c**) total soluble sugar of two soybean varieties (XD-18 and HD-19) against chromium (Cr) (0 and 100 µM) stress. Values are the means ± SD of four independent replicates. Different alphabetical letters on bars indicate significant differences among the treatments (*p* < 0.05) by the least significant difference test.

Under control conditions, there were no substantial alternations in the values of EL, TTS, and TSP among the alone treatment of BRs, and water. Although, a noticeable increase was observed in the values of EL under Cr disclosure by 57.14/42.63% in XD-18/HD-19, correspondingly (Figure 4a). Remarkably, the BRs foliar spray caused a clear reduction in the values of EL in both XD-18/HD-19 soybean cultivars by 23.14/31.67%, respectively. In the case of TSS and TSP, the Cr alone treatment caused inhibition in the values of TSS 47.05/33.33%, and TSP 68.14/52.37%, individually in XD-18/HD-19 seedlings (Figure 4b,c). Whereas, the supplementation of BRs caused a significant increment in both TSS, and TSP values by 17.98/31.76%, and 37.07/43.84% in XD-18/HD-19, correspondingly to control treatment under Cr stress (Figure 4b,c). Inclusive, these results proposed that the BRs reduced the Cr-induced stress by regulating the EL, TSS, and TSP level in both soybean genotypes, especially in HD-19.

### 2.7. BRs Decreases the ROS-Induced Cellular Oxidative Damage and Maintains Membrane Integrity by Lowering Chromium Stress

**Figure 5:**
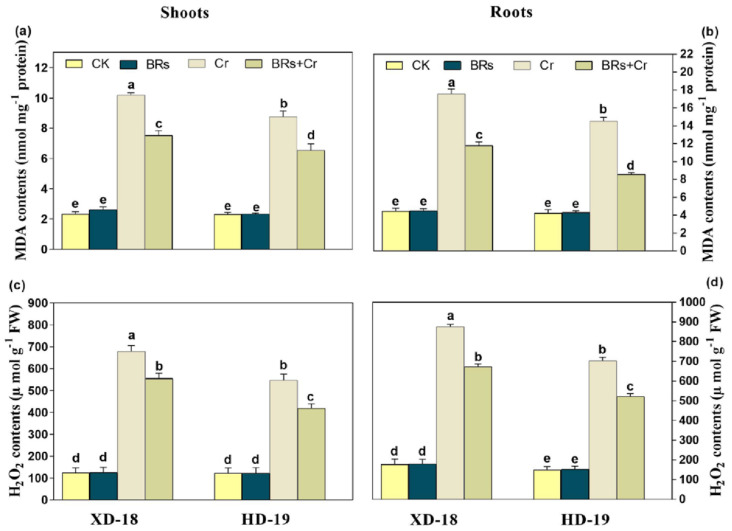
Effects of foliar-applied BRs on (**a**) MDA contents in shoots, (**b**) MDA contents in roots, (**c**) H_2_O_2,_ contents in shoots, and (**d**) H_2_O_2,_ contents in roots of two soybean genotypes (XD-18 and HD-19) under/without chromium (Cr) (0 and 100 µM) stress. Values are the means ± SD of four independent replicates. Different alphabetical letters on bars indicate significant differences among the treatments (*p* < 0.05) by the least significant difference test.

**Figure 6:**
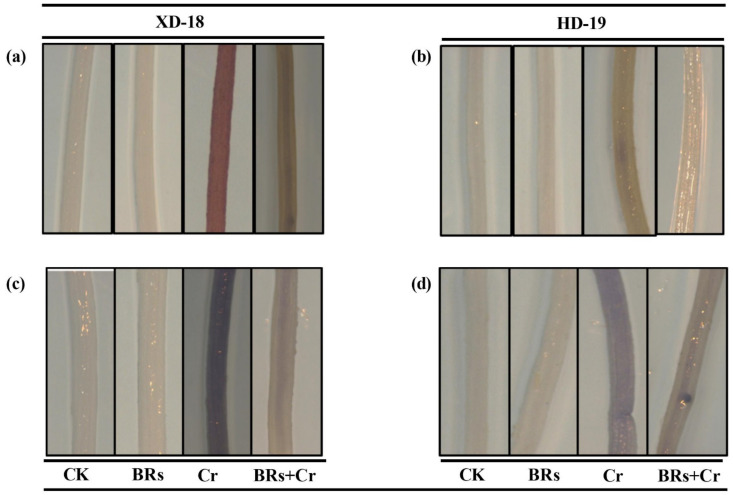
Effects of foliar-applied BRs on the histochemical staining to identify the accumulation of (**a**) hydrogen peroxide (H_2_O_2_) in the roots of XD-18, (**b**) hydrogen peroxide (H_2_O_2_) in the roots of HD-19 by 3, 3-diaminobenzidine (DAB), (**c**) superoxide (O_2_^•−^) in the roots of XD-18, and (**d**) superoxide (O_2_^•−^) in the roots of HD-19 by nitro blue tetrazolium (NBT) against chromium (Cr) (0 and 100 µM) stress.

Under Cr alone treatment, a prominent increase was observed in the values of MDA, and H_2_O_2_, contents in both shoots/roots of XD-18 by 61.89/74.97%, and HD-19 by 49.67/54.23%, respectively in comparison to the control. Conversely, the BRs application restricted the accumulation of oxidative markers in leaves/roots via inhibiting the MDA (33.59/27.92% in XD-18, and 46.70/35.86% in HD-19), as well as H_2_O_2_ (26.72/21.17%, in XD-18, and 31.78/25.78% in HD-19) level with maximum efficiency under Cr-induced stress as compared to the alone treatment of Cr (Figure 5a–d). However, there was no significant alteration in the values of MDA, and H_2_O_2_ contents level under a controlled environment (Figure 5a–d). Besides, the 3, 3-diaminobenzidine (DAB), and nitro blue tetrazolium (NBT) staining were used to further verify the H_2_O_2_ and O_2_^•−^ accumulation inside roots with/without BRs foliar application under Cr-stress in both soybean cultivars. Relative to the control, the roots of Cr treated roots represented dark brown as well as dark blue staining for H_2_O_2_ and O_2_^•−^ accumulation, respectively (Figure 6a–d). Additionally, the XD-18 soybean genotype displayed more dark staining colors than HD-19, which indicates that XD-18 accumulates more H_2_O_2_ and O_2_^•−^ as compared to HD-19. Whereas, the roots under control conditions exhibited a slight staining intensity of DAB and NBT in both soybean varieties. In general, these results have defined that MDA contents, and ROS production level were higher in roots of both soybean cultivars than shoots, and BRs supply remarkably lowers the accumulation of ROS by constraining the Cr-induced oxidative damages and sustaining the membrane integrity in both soybean genotypes, but HD-19 being more positively affected.

### 2.8. BRs Regulates Plant Antioxidants Enzymatic and Non-Enzymatic Activities under Chromium Stress

**Figure 7:**
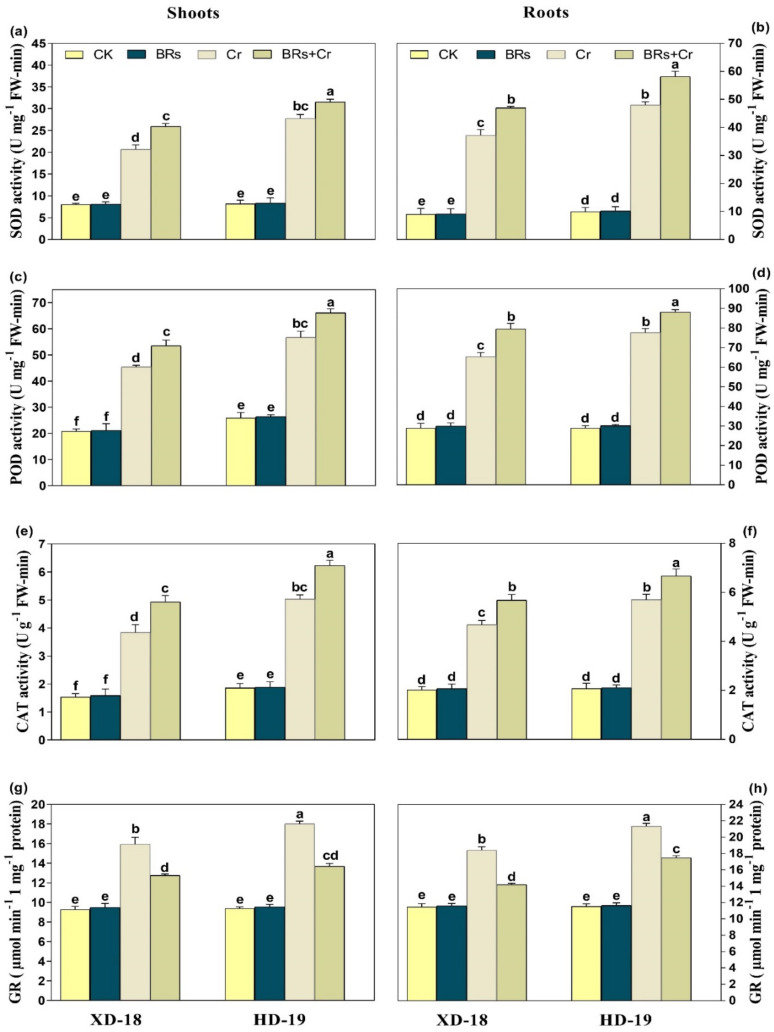
Effects of foliar-applied BRs on the activities of (**a**) superoxide dismutase (SOD) in shoots, (**b**) superoxide dismutase (SOD) in roots, (**c**) peroxidase (POD) in shoots, (**d**) peroxidase (POD) in roots (**e**) catalase (CAT) in shoots, (**f**) catalase (CAT) in roots, (**g**) glutathione (GR) in shoots, and (**h**) glutathione (GR) enzymes in the roots of both soybean genotypes (XD-18 and HD-19) under chromium (Cr) (0, and 100 µM) stress. Values are the means ± SD of four independent replicates. Different alphabetical letters on bars indicate significant differences among the treatments (*p* < 0.05) by the least significant difference test.

**Figure 8:**
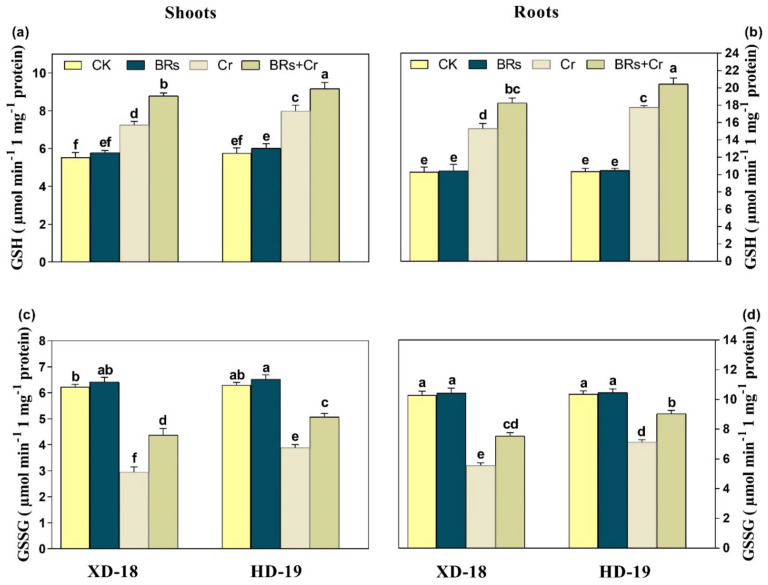
Effects of foliar-applied BRs on the activities of (**a**) GSH in shoots, (**b**) GSH in roots, (**c**) GSSG in shoots, and (**d**) GSSG in the roots of both soybean genotypes (XD-18 and HD-19) under chromium (Cr) (0, and 100 µM) stress. Values are the means ± SD of four independent replicates. Different alphabetical letters on bars indicate significant differences among the treatments (*p* < 0.05) by the least significant difference test.

Plant upsurge the antioxidative defense mechanism to overcome the deleterious effects of HMs stress. As shown in Figure 7, Cr exposure caused alternations in antioxidative enzyme activities in a recent study. Relative to the control treatment, an obvious increase was observed in the values of SOD, POD, CAT, and GR in both roots/shoots of soybean cultivar XD-18 by 60.63/72.67%, 53.33/57.38%, 43.52/58.59%, and 38.89/45.32% as well as HD-19 by 70.37/78.72%, 63.57/71.03%, 52.21/63.15%, and 47.61/55.65%, respectively under Cr disclosure. Remarkably, the BRs exposure has further stimulated the above-mentioned antioxidative enzyme activities level inside the shoot/root by 23.33/30.80%, 25.14/29.37%, 22.45/27.85%, 24.92/32.83%, respectively in XD-18, and 32.06/38.96%, 35.15/42.36%, 28.87/34.54%, and 31.48/39.04%, correspondingly in HD-19 as compared to the alone treatment of Cr under Cr-induced stress (Figure 7a–d). As well, the same trend was observed in the values of antioxidative non-enzyme activities such as the level of GSH values upsurge in shoot/roots of XD-18 by 23.61/33.33%, and HD-19 by 29.95/38.79%, respectively under alone exposure of Cr as compared to the control plants (Figure 8a). While, BRs treatment further improved the GSH level by 18.32/22.97%, and 36.84/28/16% in shoot/root of XD-18 and HD-19, respectively in comparison to control under Cr stress (Figure 8a). In contrast, the level of GSSG was decreased under Cr stress relative to its control by 54.68/68.79%, and 29.76/41.53%, individually in shoot/root of XD-18, and HD-19 (Figure 8b). Although, BRs application improved GSSG levels by 23.67/29.76%, and 33.27/41.53%, correspondingly in shoot/root of XD-18, and HD-19 as compared to the control treatment under Cr-induced toxicity (Figure 8b). Whereas, under the control environment, no significant variations were noticed in the values of antioxidant enzymatic and non-enzymatic activities of both soybean genotypes. Overall, our results indicated that Cr-induced stress caused more alternations roots of both XD-18, and HD-19 genotypes than shoots concerning the level of antioxidant enzymatic, and non-enzymatic activities, but XD-19 was more affected. However, BRs supplementation has modulated the antioxidant enzymatic, and non-enzymatic activities level in shoots as well as roots of both soybean varieties, especially in HD-19.

### 2.9. BRs Regulates Stress Responsive Genes under Chromium Stress

**Figure 9:**
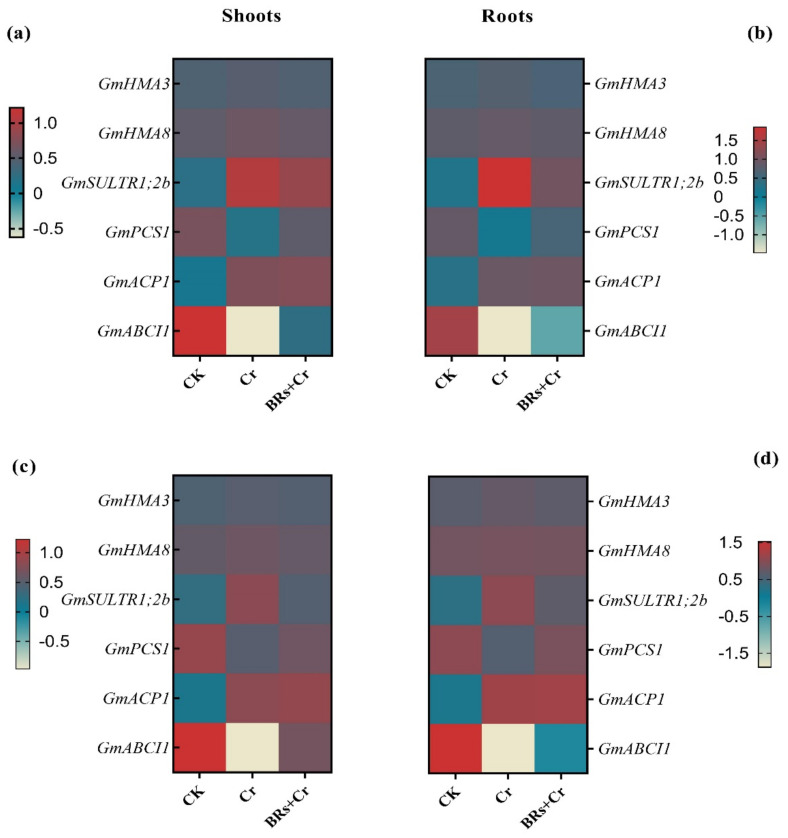
Effect of BRs foliar application on the relative expression of soybean P-type heavy metal ATPase (HMA), Sulfate (S) transporters (SULTRs), Phytochelatins (PCs), Acid−Phosphatase−Encoding Gene (*GmACP1*), and ATP binding cassette transporter (ABC) in (**a**) XD-18 shoots, (**b**) XD-18 roots, (**c**) HD-19 shoots, and (**d**) HD-19 roots of soybean genotypes under chromium (Cr) (0 and 100 µM) stress. The transcription levels were normalized with Actin (*ACT11*) in each sample.

The expression level of epitomized genes related to the translocation of Cr in two different soybean cultivars was assessed inside both roots and shoots. Inside the roots of soybean cultivars, *GmSULTR1*;*2b* may be involved in Cr uptake (Figure 9). The expression level of *GmSULTR1*;*2b*, *GmABCI1*, and *GmACP1* genes were up-regulated under Cr stress as compared to the control in both genotypes of soybean (XD-18 and HD-19). In contrast, *GmSULTR1*;*2b* expression level was significantly lowered, when BRs were sprayed to Cr-induced toxicity in comparison to the Cr-alone treated plants (Figure 9). The *GmABCI1* gene might be responsible for the ionic translocation in soybean genotypes due to its identification as well as localization in the plasma membrane inside the soybean. However, no significant difference was observed in the mRNA level of *GmHMA3*, and *GmHMA8* genes in both roots, and shoots of soybean cultivars (Figure 9a–d). In addition, the expression pattern of the *GmPCS1* gene was higher under Cr stress, when BRs were foliar-applied. This gene was identified as inorganic phosphate transporter precisely up-regulated in roots, and it may be involved in the biosynthesis of GSH which is a derivative of PCs and have a great role in Cr detoxification or/and sequestration in soybean genotypes. In general, the gene transcriptional regulation level in roots was alike to the shoots in BRs-mediated alleviation of Cr-induced toxicity.

## 3. Discussion

Being a well-known phytohormone BRs plays a considerable role to alleviate the various biotic and abiotic stresses. As Cr is a hazardous element for plant development [10,26,27], the current investigation aimed to identify the potential role of BRs and its regulation mechanism against Cr-induced damages in soybean cultivars. As per our outcomes, the excessive uptake of Cr causes severe inhibition in seed germination indices as well as growth, and biomass, which further diminishes the fresh, and dry weight, chlorophyll pigments level, total soluble sugar, and protein contents of soybean seedlings. Although, it causes significantly higher production of electrolyte leakage, MDA contents, and ROS under Cr stress. Moreover, the negative effect of Cr was more clear in XD-18 (sensitive) than HD-19 (tolerant) soybean cultivars. Interestingly, foliar application of BRs significantly enhanced the seed germination ration, photosynthetic attributes, TSS, and TSP by reducing the electrolyte leakage, MDA contents, and ROS over-generation under Cr-induced oxidative damages, and regulated the plant antioxidative defense mechanism. The mitigation effect was more prominent in HD-19 as compared to XD-18. Hence, a recent study demonstrated that BRs exogenous foliar application can efficiently mitigate the injurious effects of Cr in two different soybean genotypes. Overall, Cr showed a clear inhibition effect in seed germination indices such as GE, GP, GI, and VI. Conversely, caused a pronounced increase in MGT (Table 1). Similar results have been found in previous investigations [10,22,23] under Cr-disclosure. It might happen because Cr is directly intact with the seed coat, and lower the sugar supply by lessening the α and β amylase activates and therefore abridging the seed germination rate [12]. Additionally, our results depicted that the Cr-stress significantly reduced the plants’ biomass (FW, DW, PL). Possibly, Cr stress constrains the availability of nutrients toward the plant’s upper-most part, which ultimately inhibits the shoot length. The identical findings were identified by Wani and Khan [34] in chickpea and Islam et al. [35] in maize plants that hindered the plants growth by restricting the nutrients uptake. However, BRs supplementation improved the plants growth, and biomass which may be associated with the regulation of nutrient uptake balance (Table 2), and carbohydrate metabolism which further alleviated the Cr-induced plant growth inhibition. A sharp decrease in plant light-harvesting components was noticed (Figure 1 and Figure 2) which may be the reason for the decline in plant biomass accumulation (Table 1).

The function of PSII is directly linked with the photosynthetic electron transport chain [36]. The Cr-induced stress may cause dysfunction of the PSII system which ultimately repressed the plant biomass attributes (FW, DW). Perhaps, BRs supplementation modulated the efficiency of the PSII system, and as a result enhanced the biomass accumulation in soybean seedlings, especially in HD-19 (Table 1). In a current study, the reduction in photosynthetic, and gas exchange attributes may be because of impairment in the chlorophyll biosynthetic pathway that may occur due to restriction of the activity of the enzyme responsible for chlorophyll biosynthesis [37,38]. Similarly, the production of the photosynthetic pigment was lessened because of inhibition of the activities of δ-aminolevulinic acid dehydratase in *Nymphaea alba* under Cr-induced stress [39]. The BRs application enhanced the photosynthetic pigments, and gas exchange indices by facilitating the light-harvesting pigments in soybean cultivars under Cr stress (Figure 1 and Figure 2). Basit et al. [6] also revealed that BRs application can improve the chlorophyll contents level by regulating the electron transport, and carbon fixation which leads to light-harvesting pigment production in rice cultivars under Cr stress. Besides, it may also happen due to improvement of respiration intensity as well as the photosynthetic rate that strengthen the chlorophyll pigments biosynthesis, and maximum quantum yield of PSII photochemistry (F*v*/F*m*) under a stressed environment [20]. Matched outcomes were noted in *T. aestivum* [40], and *B. juncea* [41] under Cd, and Cr-induced toxicity.

Relative to the control, a greater accumulation of Cr contents was noticed in roots than shoots of both soybean cultivars, particularly in XD-18 (Table 2). Hence, it displayed that the Cr higher uptake occurred by roots which further transported to the top-most parts of plants. The greater Cr contents level in roots and slight translocation from roots toward shoots supported the aforementioned results [38,42]. However, BRs supplementation has restricted the Cr uptake as well as translocation from roots to shoots. Possibly, BRs have increased the metal ion binding contents, i.e., pectin and hemicelluloses inside the cell wall of the root which was responsible for a higher concentration of Cr in the root cell wall [12,43]. Generally, BRs presence may be essential for pectin and hemicelluloses to bind the Cr ion with the root cell wall, and thus restrict the intercellular distribution of Cr in soybean seedlings. Regarding the nutrient uptake, Cr stress caused a significant reduction. Cr excessive level may replace the nutrient elements such as Ca, Mg, Mn, Zn, and Cu due to its ionic resemblance, and resultant enhanced Cr translocation toward shoots [44]. Recently, this nutrient imbalance was higher in roots in comparison to shoots of both soybean genotypes, with XD-18 being more negatively affected (Table 2). Whereas, BRs improved the nutrient uptake balance most probably by reducing the membrane damage induced by Cr-induced higher ROS production, electrolyte leakage, and lipid peroxidation. Likewise, the same results have been demonstrated by Basit et al. [45] in rice, and Choudhary et al. [46] in radish. In the latest research, control under Cr exposure significantly up-surged ROS over-production, and lipid peroxidation (Figure 5 and Figure 6). It perhaps happened due to the induction of cellular damage inside plant tissues under Cr toxicity [47]. The lipid peroxidation may have a direct link with photosynthetic pigments level which might have persuaded the ROS extra-accumulation when Cr came in contact with the electron transport chain carrier [48], which further disturbed the membrane stability. Although, BRs foliar treatment provided membrane integrity as well as stability by reducing the ROS over-production, and MDA contents level to minimize the Cr-induced stress damage under Cr disclosure (Figure 5 and Figure 6). Similar results are revealed under Cr stress in tomato [49], rice [6], and radish [46].

Plants provoke their antioxidative defense mechanism to alleviate the Cr-induced cellular oxidative damage by reducing ROS excessive production. Current investigation has displayed that plants up-surged the antioxidant enzymatic (SOD, POD, CAT, GR), and non-enzymatic activities GSH under Cr stress. In contrast, GSSG activity is reduced under Cr disclosure (Figure 7 and Figure 8). The modulation in antioxidative enzyme, and non-enzymatic activities were also shown in *B. oleracea* [50], *Oryza stiva* [7], *Z. mays* [35], and *Triticum aestivum* [51]. Moreover, exogenous application of BRs detoxifies the Cr-induced oxidative stress, and maintained the redox balance by further stimulating the antioxidant enzymatic, and non-enzymatic activities (Figure 7 and Figure 8). Earlier investigations also supported our outcomes that BRs supplementation further improved the level of antioxidative activities under numerous stresses such as Cr [48,52], Al [45], Cd [53], and Mn [54].

The uptake, accumulation, and translocation of Cr in soybean persuaded by foliar applicator of BRs might be related to the effect of BRs on the expression pattern of a few related genes that may be intricate in Cr translocation, i.e., *GmSULTR1;2b*, *GmABCI1*, and *GmACP* (Figure 9). Although, to mitigate the Cr-induced phytotoxicity, foliar-applied BRs probably metastasized to the cytoplasm by *GmPCS1* and chelated with Cr to form a complex [4,55]. Consequently, the PCs-Cr complex may be accumulated inside chloroplasts, vacuoles, or translocated to the shoot by some transporters. As a result, Cr accumulation, sequestration as well as the diminished concentration of the soluble fraction happened. In a current study, *GmPCS1* was upregulated, when BRs were applied to both soybean cultivars (Figure 9). Likewise, previous studies also supported our results that PCs play a crucial role to improve the plants’ tolerance against numerous heavy metal toxicities [4,56,57].

## 4. Materials and Methods

### 4.1. Plant Materials

Two different soybean cultivars Xudou-18 (XD-18) as a sensitive cultivar, and Huaidou-19 (HD-19) as a tolerant cultivar were purchased from the Zhejiang Nongke Seeds CO., LTD. Hangzhou, Zhejiang Province, China for the recent investigation.

### 4.2. Seed Germination Analysis

The sterilization of seeds was carried out by dipping into 5% (*w*/*v*) sodium hypochlorite (NalCO) solution for 20 min, and rapidly eroded with double-distilled dd H_2_O to get rid of enduring chloride. Later, attained seeds were germinated to evaluate the germination test analysis. All germination boxes were placed in a growth chamber at 25 °C with a variation cycle of 8/16 h illumination/dark conditions for 14 days, correspondingly [58]. The incubated seeds were sprayed with 0, and 0.01µM BRs along with exposure to 0 and 100 µM chromium stress. The calculation of germinated seeds was conducted out on a daily basis for sequential first 14 days. The percentage (%) of germinated seeds on the 5th day was deliberated as germination energy (GE), and the % of seeds germinated at the end of the complete test on 14th day was known as germination percentage (GP). Germination index (GI), mean germination time (MGT) and vigor index (VI) were assessed by following Zheng et al. [58].

GI = Σ(Gt/Tt)(1)

MGT = Σ(Gt × Tt)/ΣGt(2)

VI = Germination % × [Shoot length + Root length](3)

Gt is the total calculated germinated seeds on day t, and Tt is the time related to Gt in days [58].

### 4.3. Hydroponic Culture, Treatments, and Growth Conditions

Inside a black hydroponic container with a diameter ~25 cm, the seven-day-old seedlings were put and filled with nutrient solution. Every treatment was repeated four times, and 50 seeds were utilized in every replication. The hydroponic containers were repositioned daily in a growth chamber with a turning cycle of 12/12 h illumination/dark with 550 µmol m^−2^ s^−1^ light intensity, and via a completely randomized design. Additionally, for every treatment four replication were selected after two weeks of hydroponics to disclose the 100 µM Cr concentration of K_2_Cr_2_O_7_ with a nutrient media solution for 7 days, and the other four repeats without Cr stress were used as control (CK). Sampling was carried out after the 7th day of Cr stress treatment.

### 4.4. Sampling, and Plant Growth Analysis

The harvesting of soybean seedlings was performed on the 21st day and washed with (dd) H_2_O to eradicate the deposits of Cr. The seedling’s height was calculated by a ruler, and their fresh mass was assessed on the measuring balance. To determine the dry masses, shoots and roots were oven-dried discretely at 70 °C for the whole day.

### 4.5. Determination of Photosynthetic, and Chlorophyll Fluorescence Measurements

The chlorophyll contents of soybean leave were measured through a portable device (SPAD-502+, Tokyo, Japan). Moreover, the gas exchange parameters i.e., net photosynthetic, transpiration rate, stomatal conductance, CO_2_ assimilation rate, and maximum quantum yield of PSII photochemistry (F*v*/F*m*) were estimated by deliberating the methodology of Schreiber and Bilger [59] to measure the seedlings with a LiCor-6400 portable photosynthesis system (Li-Cor Inc., Lincoln, NE, USA). However, the calculations for the chlorophyll fluorescence were performed according to Zivcak, and Kalaji [60,61]. Meanwhile, 2 h of acclimatization inside the growth cabinet at 18 °C, 1000 mol m^−2^ s^−1^ light intensity, and 60% comparative moisture, the topmost entirely extended leaves were selected, and ImagingWin software (IMAGING-PAM, Walz, Effeltrich, Germany) was used to inspect the colored images for F*v*/F*m* and F*m* levels.

### 4.6. Determination of Cr Accumulation, and Micronutrients Level

Almost 0.2 g ground dried samples of plant roots, and shoots were utilized to estimate the concentrations of Cr contents in soybean seedlings. The samples were heated on a hot plate at 500 °C for 24 h, and then assimilated in HNO_3_-HClO_4_ (3:1, *v*/*v*) for 48 h at room temperature. Afterward, the solution of digested samples was diluted with 15 mL dd H_2_O. The final filtrate was used to quantify the Cr and microelements Ca, Mg, Zn, Cu, and Mn contents measurements by using an atomic absorption spectrometer [62].

Translocation factors (TFs) = [(Shoot Cr/Root Cr) × 100](4)

### 4.7. Determination of Total Soluble Sugar, Protein, and Electrolyte Leakage

To estimate the electrolyte leakage as EL (dSm^−1^), 1 g seedlings were speckled with ddH_2_O using at least four repeats. Later, seedlings were dipped into the 25 mL dd H_2_O and incubated (25 °C) for 24 hrs. The samples were transferred to another blank beaker and dd H_2_O was added up to 25 mL volume, EL was quantified in dSm^−1^ conferring to the previous protocol [63]. To determine the total soluble sugar (TSS), fresh leaf samples of 0.5 g of soybean seedlings were ground in mortars and pestles along with an extraction buffer in it. Phosphate buffer (50 mM, pH 7), glycerol (10%, *v*/*v*), ascorbate (1 mM), potassium chloride (100 mM), and β-mercaptoethanol (5 mM) was used to prepare the extraction buffer. After, the homogenate was amassed into the micro-centrifuge tube by 15 min centrifugations at 12,000× *g*. Well along, the precipitate was further utilized for quantification of TSS, followed by phenol-sulfuric acid assay [64]. Moreover, the supernatant was used to analyze the total soluble protein (TSP) as per the method of Bradford [65].

### 4.8. Determination of Malondialdehyde Level, and Hydrogen Peroxidation Production

The investigation of MDA content was performed with 2-thiobarbituric acid (TBA), a fresh sample (0.5 g) was used to extract tissues of 1.5 mL, and was homogenized in 2.5 mL of 5% TBA and diluted in 5% trichloroacetic acid. Far along, homogenized samples were heated at 90 °C for 20 min and formerly cooled at ice immediately, and centrifuged at 532 and 600 nm wavelengths using UV–vis spectrophotometer (Hitachi U-2910) [66]. The value of MDA content was expressed as nmol mg^−1^ protein.

The hydrogen peroxide (H_2_O_2_) was estimated by homogenizing the plant tissues in phosphate buffer and centrifuged at 6000 g. The 0.1% titanium sulfate containing 20% (*v*/*v*) H_2_SO_4_ was added to obtain supernatant and intermixed. The intensity of the yellow color was measured calorimetrically at 410 nm [56]. The contents of H_2_O_2_ were quantified by constructing the standard curve with the known concentration of H_2_O_2_ and a reaction mixture without plant tissue was considered as control along with its observations subtracted from treatments. The H_2_O_2_ contents were calculated in terms of (µmol g^−1^ FW) at 25 ± 2 °C.

### 4.9. Antioxidative Enzymatic, and Non-Enzymatic Assay

The samples of uppermost expended leaves and roots were used to homogenize in particular buffers under ice-cold conditions for each antioxidant. To estimate the SOD activity, the absorbance discrepancies of the reaction mixture counting 50 mM KH_2_PO_4_ buffer, 2.24 mM NBT (nitro blue tetrazolium), 0.1 units xanthine oxidase, and 2.36 mM xanthine were testified for 2 min according to the described protocol of El-Shabrawi et al. [57]. The required amount of enzyme was used to restrict NBT reduction (50%), and one unit of SOD activity was evaluated as a unit (min^−1^ mg^−1^ protein). The catalase (CAT) activity was determined by following the methodology of Hossain et al. [67], and used as 39.4 M^−1^ cm^−1^ of extinction coefficient. In addition, POD activity was estimated as per the method of Change and Maehly [68], and the enzyme activities were calculated in terms of l M of guaiacol oxidized g^−1^ FW min^−1^ at 25 ± 2 °C. However, the non-enzymatic activities i.e., GR was assayed as per the method of Schaedle and Bassham [69] with a slight modification as well as GSH, and GSSG were measured according to the protocol of Law et al. [70].

### 4.10. Determination of Cr Stress-Responsive Gene Expression Analysis

Quantitative real-time PCR (qRT-PCR) was performed to analyze the gene expression of Cr stress-responsive (SLUTRs, HMA, PCs, etc.). The samples of both shoots and roots were ground in liquid nitrogen carefully by using a mortar, and pestle. Trizol mixture was used to extract the total RNA of soybean seedlings according to the defined protocol of Sah et al. [71]. The concentration and purity of RNA were quantified by NanoDrop 2000/2000c (Thermo Scientific, Waltham, WA, USA). In addition, to synthesize the cDNA, The PrimeScript™ RT reagent kit was used. Primers used in this study are given in Table S1. The relative alternations in gene expression patterns were analyzed followed by Livak and Schmittgen [72] methodology. To set the internal calibration as a control gene, *GmActin* was used to 250 and normalize the other genes.

### 4.11. Statistical Analysis

All experimental data were analyzed by applying analysis of variance with the least significant differences (LSD) at *p* < 0.05 and *p* < 0.01 levels between mean values using Statistix software version 8.1. All the experiments were repeated at least four times.

## 5. Conclusions

In a nutshell, foliar application of BRs lowers the Cr uptake, and translocation in soybean cultivars by regulating the relative expression of genes associated with Cr absorption, and further activates the phytochelatins to detoxify the Cr accumulation in both soybean cultivars (XD-18, and HD-19). As a result, improve the accumulation of plant biomass, photosynthetic pigment level, total soluble sugar, and protein to maintain the balance of electrolytes, and mineral nutrients under Cr stress in soybean cultivars. In addition, BRs supplementation stimulates the antioxidative defense system to identify, and scavenge the extra ROS generation. Thus, BRs may be involved in signal transmission by their morphological proportion. Our findings were significant for the foliar application of BRs to enhance the soybean bioremediation on Cr-polluted land. However, field trials with a cost-to-benefit ratio should be scrutinized to endorse the BRs usage as a Cr-toxicity antinode for abating the yield losses in Cr-polluted soils.

## Figures and Tables

**Table 1:** Foliar application of BRs regulates the various germination and growth parameters in two different soybean genotypes such as germination index (%), germination percentage (%), mean germination time (%), biomass (fresh-FW and dry-DW weights), and plant length (PL) with/without Cr stress conditions.

Genotypes	Varieties Name	GE	GP	GI	MGT	VI	FW	DW	PL
XD-18	CK	98.67 ± 2.31a	98.00 ± 2.00a	35.40 ± 0.33b	2.05 ± 0.05e	4.03 ± 0.31a	0.89 ± 0.02b	0.11 ± 0.00b	29.91 ± 0.22b
	BRs	99.67 ± 1.15a	100.33 ± 1.15a	36.04 ± 0.46ab	1.92 ± 0.03ef	4.33 ± 0.25a	0.92 ± 0.02a	0.12 ± 0.00ab	30.92 ± 0.37ab
	Cr	26.00 ± 2.00e	27.33 ± 1.15e	7.07 ± 0.04f	2.71 ± 0.19a	0.43 ± 0.01e	0.23 ± 0.04f	0.03 ± 0.00f	13.59 ± 0.41f
	BRs + Cr	55.33 ± 1.15c	56.00 ± 0.00c	18.79 ± 0.63d	2.11 ± 0.08cd	1.52 ± 0.31 c	0.55 ± 0.01d	0.08 ± 0.00d	23.64 ± 0.13d
HD-19	CK	99.67 ± 2.31a	99.63 ± 1.15a	35.71 ± 0.19b	1.93 ± 0.04ef	4.29 ± 0.19a	0.91 ± 0.03ab	0.12 ± 0.00ab	30.25 ± 0.29ab
	BRs	100.00 ± 0.00a	100.00 ± 0.00a	36.67 ± 0.34a	1.85 ± 0.02f	4.41 ± 0.14a	0.93 ± 0.21a	0.13 ± 0.00a	31.49 ± 0.35a
	Cr	46.00 ± 2.00d	46.67 ± 1.15d	15.03 ± 0.71e	2.32 ± 0.10b	1.03 ± 0.09d	0.46 ± 0.02e	0.05 ± 0.00e	19.52 ± 0.08e
	BRs + Cr	64.00 ± 2.00b	64.00 ± 2.00b	22.99 ± 0.20c	2.10 ± 0.14d	1.93 ± 0.27b	0.68 ± 0.12c	0.09 ± 0.00cd	25.78 ± 0.29c

The same letters within a column designate there was no significant difference at a 95% probability level at the *p* < 0.05 level according to the LSD test, correspondingly.

**Table 2:** Effect of foliar-applied BRs on the accumulation of endogenous calcium (Ca), magnesium (Mg), manganese (Mn), zinc (Zn), and copper (Cu) ions in the shoots, and roots of two soybean cultivars (XD-18 and HD-19) under Cr stress.

Varieties	Treatments	Ca	Mg	Mn	Zn	Cu
		Shoots (mg/g)				
XD-18	CK	1.53b ± 0.47	0.41a ± 0.00	1.89c ± 0.56	0.47a ± 0.08	0.07a ± 0.003
	BRs	1.58ab ± 0.18	0.42a ± 0.01	1.95ab ± 0.24	0.49a ± 0.06	0.07a ± 0.002
	Cr	0.39f ± 0.13	0.36a ± 0.03	0.31g ± 0.67	0.19e ± 0.01	0.02 ± 0.001
	BRs + Cr	1.09d ± 0.17	0.38a ± 0.02	0.71e ± 0.31	0.29cd ± 0.01	0.05a ± 0.002
HD-19	CK	1.57ab ± 0.78	0.41a ± 0.02	1.93bc ± 0.33	0.48a ± 0.03	0.07a ± 0.002
	BRs	1.61a ± 0.58	0.43a ± 0.01	1.97a ± 0.72	0.49a ± 0.07	0.08a ± 0.010
	Cr	0.78e ± 0.14	0.38a ± 0.02	0.43f ± 0.20	0.27d ± 0.04	0.04a ± 0.001
	BRs + Cr	1.14cd ± 0.32	0.40a ± 0.04	0.74de ± 0.49	0.39b ± 0.04	0.05a ± 0.001
**Varieties**	**Treatments**	**Roots (mg/g)**				
XD-18	CK	0.39a ± 0.01	0.53a ± 0.05	0.34a ± 0.03	0.33b ± 0.03	0.09a ± 0.02
	BRs	0.39a ± 0.00	0.54a ± 0.02	0.35a ± 0.07	0.36ab ± 0.04	0.10a ± 0.07
	Cr	0.17c ± 0.03	0.22d ± 0.01	0.09e ± 0.01	0.06e ± 0.01	0.02d ± 0.05
	BRs + Cr	0.36a ± 0.01	0.35b ± 0.03	0.27c ± 0.05	0.28c ± 0.11	0.06b ± 0.03
HD-19	CK	0.39a ± 0.02	0.53a ± 0.03	0.35ac ± 0.04	0.35ab ± 0.04	0.10a ± 0.03
	BRs	0.40a ± 0.01	0.55a ± 0.02	0.36a ± 0.09	0.38a ± 0.06	0.10a ± 0.05
	Cr	0.31b ± 0.01	0.29c ± 0.01	0.21d ± 0.01	0.18d ± 0.01	0.04c ± 0.01
	BRs + Cr	0.37a ± 0.02	0.37b ± 0.02	0.29bc ± 0.17	0.30bc ± 0.30	0.07ab ± 0.02

The same letters within a column designate there was no significant difference at a 95% probability level at the *p* < 0.05 level according to the LSD test, correspondingly.

## Data Availability

Data can be requested from the corresponding authors.

## Supplementary Materials

The following supporting information can be downloaded at: https://www.mdpi.com/article/10.3390/plants11172292/s1, Figure S1. Effect of EBL foliar application on the visual appearance of soybean seedlings under chromium (Cr) toxicity, Table S1. Primers information, Table S2. Foliar application of BRs improves the chlorophyll fluorescence level such as net photosynthetic rate (Pn), transpiration rate (Tr), stomatal conductance (gs), CO_2_ assimilation rate (Ci), and maximum quantum yield of PSII photochemistry (F*v*/F*m*) in two different soybean cultivars under chromium (Cr) stress.
