# Correction: Basit et al. Brassinosteroid Supplementation Alleviates Chromium Toxicity in Soybean (*Glycine max* L.) via Reducing Its Translocation. *Plants* 2022, *11*, 2292

**DOI:** 10.3390/plants15132040

**Published:** 2026-07-01

**Authors:** Farwa Basit, Javaid Akhter Bhat, Jin Hu, Prashant Kaushik, Ajaz Ahmad, Yajing Guan, Parvaiz Ahmad

**Affiliations:** 1 The Advanced Seed Institute, College of Agriculture and Biotechnology, Zhejiang University, Hangzhou 310058, China; 11916138@zju.edu.cn (F.B.); jhu@zju.edu.cn (J.H.); 2 International Genome Center, Jiangsu University, Zhenjiang 212013, China; javid.akhter69@gmail.com; 3 Independent Researcher, 46022 Valencia, Spain; prakau@doctor.upv.es; 4 Department of Clinical Pharmacy, College of Pharmacy, King Saud University, Riyadh 11451, Saudi Arabia; ajukash@gmail.com; 5 Department of Botany, GDC Pulwama, Srinagar 192301, Jammu and Kashmir, India

In the publication [1], there was an error regarding the affiliation for Prashant Kaushik. The updated affiliation should be as follows: Independent Researcher, 46022 Valencia, Spain.

The authors state that the scientific conclusions are unaffected. This correction was approved by the Academic Editor. The original publication has also been updated.
