# Supplementary material for: Brassinosteroid Supplementation Alleviates Chromium Toxicity in Soybean (Glycine max L.) via Reducing Its Translocation
Source: Plants (Basel). 2022 Sep 1;11(17):2292. doi: 10.3390/plants11172292 (PMC9460071; doi:10.3390/plants11172292)
Supplement: Supplementary file 1 [file plants-11-02292-s001.zip › plants-1865028-supplementary.pdf]

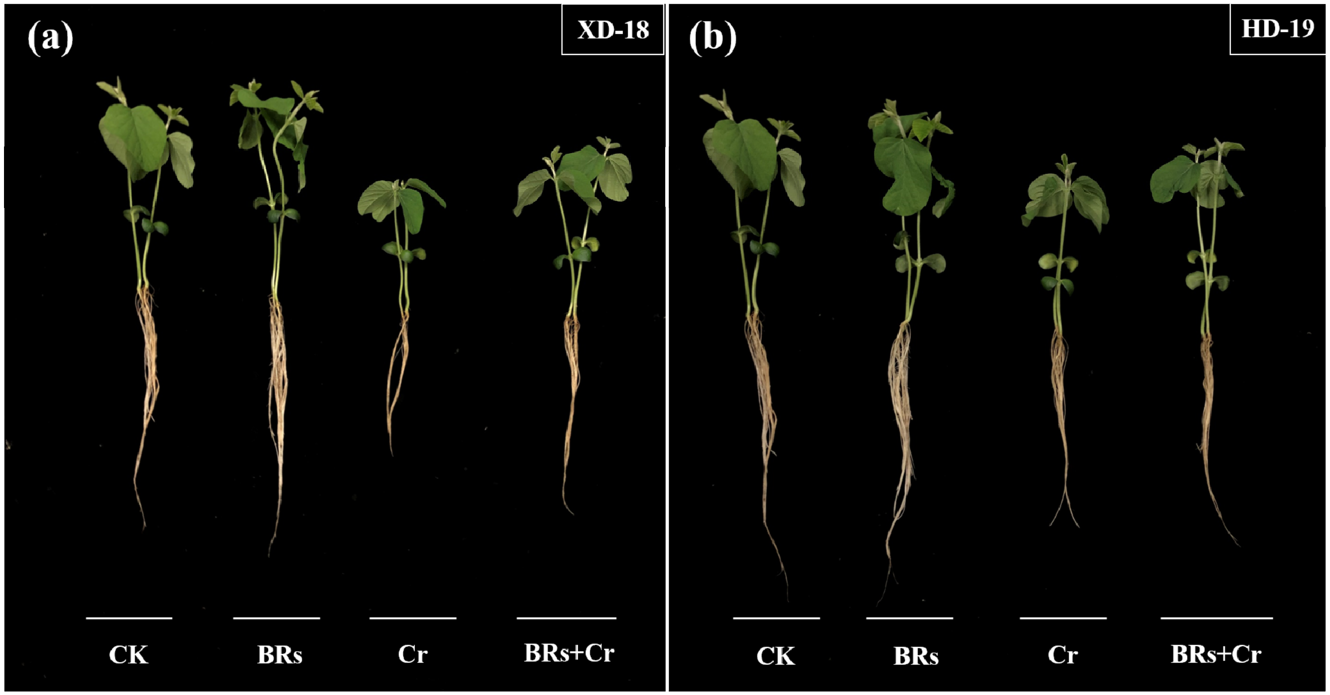

Figure S1. Effect of EBL foliar application on the visual appearance of soybean seedlings under chromium (Cr) toxicity. (a) Cr-induced phenotypical changes on the XD-18 cultivar and mitigation effect of EBL under Cr stress. (b) Cr-induced phenotypical changes on the HD-19 cultivar and mitigation effect of EBL under Cr exposure.

Table S1. Primers information

Gene Name	Description	Forward primer	Reverse primer
GmHMA3	Zinc-transporting ATPase	GCGCTTAGAAGAATTGA AGGGAT	GCCTTGCTTGATTCAGTGCC
GmHMA8	Chloroplast copper-translocating P-ATPase	CGCTCTGAGACTGAGCG ATT	CTCCATAAGCGGTCCGTGAG
GmSULTR1;2b	High-affinity sulfate transporter	GGTCTTCTAATTGCGGT GGC	TGACGATCCACTTCTCCTTT GG
GmhPCS1	Phytochelatin synthase 1 (PCS1)	AAGGCTTGCTATCAAGG AAGAGG	CTGATACTTGTTGCCTGCTG C
ACT11	Cytoskeletal structural protein	ATCTTGACTGAGCGTGG TTATTCC	GCTGGTCCTGGCTGTCTCC

Table S2. Foliar application of BRs improves the chlorophyll fluorescence level such as net photosynthetic rate (Pn), transpiration rate (Tr), stomatal conductance (gs), CO_2 assimilation rate (Ci), and maximum quantum yield of PSII photochemistry (F_v/F_m) in two different soybean cultivars under chromium (Cr) stress

Genotypes	Treatments	Net photosynthetic rate	SPAD values	Transpiration rate	Stomatal conductance	CO_2 assimilation rate	F_v/F_m
XD-18	CK	8.95±0.12a	34.7±1.43b	5.37±0.11b	0.36±0.02b	433.67±5.23b	0.56±0.02c
	BRs	9.01±0.18a	35.6±1.08ab	5.49±0.09ab	0.38±0.16a	435.97±8.07ab	0.59±0.01bc
	Cr	3.89±0.14e	13.6±0.78f	2.01±0.08f	0.16±0.01f	202.43±11.38e	0.27±0.02g
	BRs+Cr	6.32±0.29c	26.8±0.23d	3.58±0.07d	0.26±0.02d	298.76±12.62d	0.43±0.01e
HD-19	CK	8.97±0.05a	35.3±0.34ab	5.55±0.16a	0.37±0.09ab	436.31±11.43ab	0.63±0.01ab
	BRs	9.04±0.11a	36.1±0.65a	5.57±0.07a	0.38±.08a	439.34±3.42a	0.66±0.03a
	Cr	5.54±0.13d	21.9±0.93e	2.91±0.11e	0.21±0.01e	256.31±6.75e	0.35±0.03f
	BRs+Cr	7.49±0.19b	29.7±1.54c	4.34±0.18c	0.29±0.03c	334.26±15.74c	0.49±0.02d

The same letters within a column designate there was no significant difference at a 95% probability level at the $p < 0.05$ level according to the LSD test, correspondingly.
